# Evolution of the CRISPR-Cas9 defence system in Mycoplasma gallisepticum following colonization of a novel bird host

**DOI:** 10.1099/mgen.0.001320

**Published:** 2024-11-18

**Authors:** Thomas Ipoutcha, Iason Tsarmpopoulos, Géraldine Gourgues, Vincent Baby, Paul Dubos, Geoffrey E. Hill, Yonathan Arfi, Carole Lartigue, Patricia Thébault, Camille Bonneaud, Pascal Sirand-Pugnet

**Affiliations:** 1 Univ. Bordeaux, INRAE, UMR BFP, F-33882, Villenave d’Ornon, France; 2 Department of Biological Sciences, Auburn University, Auburn, Alabama, 36849-5414, USA; 3 Univ. Bordeaux, CNRS, Bordeaux INP, LaBRI, UMR 5800, F-33400 Talence, France; 4 Centre for Ecology and Conservation, University of Exeter, Penryn TR10 9FE, UK; 5 Environment and Sustainability Institute, University of Exeter, Penryn TR10 9FE, UK

**Keywords:** bird pathogen, CRISPR-Cas, evolution, host shift, mycoplasma

## Abstract

Clustered regularly interspaced short palindromic repeat (CRISPR)-Cas systems are bacterial defences that target bacteriophages and mobile genetic elements. How these defences evolve in novel host environments remains largely unknown. We studied the evolution of the CRISPR-Cas system in *Mycoplasma gallisepticum* (also named *Mycoplasmoides gallisepticum*), a bacterial pathogen of poultry that jumped into a passerine host ~30 years ago. Over the decade following the host shift, all isolates displaying a functional CRISPR-Cas system were found not only to harbour completely new sets of spacers, but the DNA protospacer adjacent motif recognized by the main effector *M. gallisepticum* Cas9 (MgCas9) was also different. These changes in CRISPR-Cas diversity and specificity are consistent with a change in the community of phages and mobile elements infecting *M. gallisepticum* as it colonized the novel host. In the years following the host shift, we also detected a gradual rise in isolates displaying non-functional MgCas9. After 12 years, all circulating isolates harboured inactive forms only. This loss of CRISPR-Cas function comes at a time when the passerine host is known to have evolved widespread resistance, which in turn drove the evolution of increasing *M. gallisepticum* virulence through antagonistic coevolution. Such striking concordance in the rise of inactivated forms of CRISPR-Cas and the evolution of host resistance suggests that the inactivation of the CRISPR-Cas system was necessary for enabling adaptive bacterial responses to host-driven selection. We highlight the need to consider both host and pathogen selection pressures on bacteria for understanding the evolution of CRISPR-Cas systems and the key factors driving the emergence of a pathogenic bacterium in a novel host.

**Impact Statement**

Mycoplasma are minimal bacteria involved in many diseases affecting humans and a wide diversity of animals. In this paper, we report the evolution of the type II clustered regularly interspaced short palindromic repeat (CRISPR)-Cas system of the bird pathogen, *Mycoplasma gallisepticum*, following a host jump from its original poultry host into its novel house finch host in the early 1990s. Instances in which bacterial pathogens have been documented to jump into and subsequently adapt to a new host are rare, and the well-documented case of *M. gallisepticum* is a unique model to evaluate the effect of any dramatic host environmental change on bacterial CRISPR-Cas defence systems. First, we performed *in silico* analyses on an extended set of 98 *M*. *gallisepticum* genomes to better understand the evolution of the CRISPR-Cas9 system in the novel finch host. We documented several evolutionary events leading to the drastic divergence of spacer sets present in poultry and house finch arrays, as well as the progressive inactivation of the CRISPR-Cas system after 12 years in the novel finch host. Second, using *in vitro* and *in vivo* assays, we demonstrated that the evolution of the *M. gallisepticum* Cas9 protospacer adjacent motif (PAM)-interacting domain, involved in the PAM recognition, has led to a major change in the defence system, with a modification of the recognized PAM in the novel host. Such radical change in the CRISPR-Cas defence system of *M. gallisepticum* may have implications for its rapid adaptation to its novel host. Together, our results highlight the need to consider not only the host-driven selection pressures a bacterium experiences but also the complex interplay between phages and defence systems for a better understanding of the key factors driving the emergence of a pathogenic bacterium in a novel host.

## Data Summary

The authors confirm that all supporting data and protocols have been provided within the article or through supplementary data files available in the online version of this article. GenBank accession numbers of all publicly available *M. gallisepticum* genomes are listed in Table S3. Sequences of the CRISPR locus of other isolates are also provided in Table S3. Supplementary material is available in the online version of this article, available through Figshare at 10.6084/m9.figshare.27165426 [1].

## Introduction

Clustered regularly interspaced short palindromic repeat (CRISPR) systems are defences found in most prokaryotes that effectively protect cells against the constant threat of phages and other sources of invading DNAs. The flexibility and effectiveness of this defence system rely on the continuous acquisition of new spacers to the CRISPR array [2], which entails substantial maintenance costs [3]. As a consequence, the inactivation and disappearance of CRISPR systems from prokaryote genomes are predicted to occur when the benefits of this defence system are relaxed [4,5]. Ultimately, gain or loss events of active CRISPR systems will be shaped both by cost/benefit ratios [6] and by the complex ecological pressures experienced by prokaryotes. For pathogenic bacteria, the strongest ecological pressures arise from interactions with hosts. Changes in host-driven selection pressures may occur, for instance, as hosts evolve in response to pathogens, but should be particularly dramatic when a prokaryotic parasite colonizes a novel host. While invasion of a novel host is known to broadly impact bacterial genomes, how such host shifts shape bacterial defence systems against phages and other parasitic DNAs remains largely unstudied.

Instances in which bacterial pathogens have been documented to jump into and subsequently adapt to a new host species are rare [7], limiting opportunities to test the effect of host environmental change on bacterial CRISPR systems. One model in which the adaptation of a bacterium to a novel host can be studied is the *Mycoplasma gallisepticum*/house finch (*Haemorhous mexicanus*) system. *M. gallisepticum* (also named *Mycoplasmoides gallisepticum*) is a bacterial pathogen that is common in domestic chickens (*Gallus gallus*), a gallinaceous bird. It jumped into a very distantly related and widely distributed North American passerine, the house finch. House finch isolates have experimentally been shown to display a reduced ability to infect chickens [7,8], with cross-species transmission occurring only under unnatural conditions of prolonged direct inter-specific contact and inoculation, showing a real host shift had occurred. This novel host shift was documented in 1994 by wildlife disease experts [9], and a mid-1990s host-shift date has been confirmed by genome analyses of *M. gallisepticum* isolates collected from chickens and finches. *M. gallisepticum* lineages subsequently collected across the range of the house finches indicate that all *M. gallisepticum* circulating in house finch populations are derived from this single host-shift event [10,11].

*M. gallisepticum* is an avian pathogen that belongs to the class *Mollicutes* [12], a group of minimal bacteria characterized by a rapid evolution through drastic genome reduction from a Gram-positive ancestor. Such genetic losses are partially counter-balanced by horizontal gene transfers (HGTs) and exposure to mobile genetic elements (MGEs) acting as potential sources of genetic diversity and genome reorganization [13,14]. Despite an evolutionary tendency towards genome minimization, most Mollicutes have retained various defence systems against phages and invading DNAs. Indeed, Mollicutes show the second highest density of restriction–modification systems [15]. They are also the free-living bacteria with the smallest genome in which CRISPR-Cas systems have been described [16].

Changes in the CRISPR locus of *M. gallisepticum* have previously been reported following the host shift into house finches [10,17]. Indeed, a comparison of *M. gallisepticum* isolates obtained from ancestral poultry and novel house finch hosts showed a complete change in the spacer set, with no elements being shared between poultry and house finch isolates. House finch isolates also displayed a loss of spacers over time, as well as a degradation of their CRISPR locus [10]. While these patterns suggest both a slowing down in the recruitment of new spacers and even an evolution towards this defence system becoming non-functional, further work is required to test whether these evolutionary changes have persisted over time in the new passerine host. A better understanding of the observed changes in the house finch CRISPR also requires the characterization of other changes that have occurred in the CRISPR-Cas system and particularly in *M. gallisepticum* Cas9 (MgCas9). Previous research has shown that Cas9 endonuclease is the main effector of type-II CRISPR systems, being involved in both the acquisition of new spacers and the double-strand DNA cleavage of target DNAs [18,19]. In both processes, Cas9 selects functional spacers by recognizing their protospacer adjacent motif (PAM) sequence immediately downstream. The change of spacer set that has accompanied *M. gallisepticum* host shift therefore suggests the evolution of MgCas9 PAM specificity as a possible driver.

To better understand the changes in the *M. gallisepticum* CRISPR system that took place during the colonization of the novel host, we conducted a functional characterization using a two-pronged approach. First, we performed *in silico* analyses on an extended set of *M. gallisepticum* isolates (79 collected from 1994 to 2015) to better understand the evolutionary changes in CRISPR-Cas9. We identified several events leading to the remarkable divergence of spacer sets in poultry versus house finch arrays and an ongoing inactivation of the CRISPR-Cas system in the house finch host over time. Second, we functionally characterized the CRISPR-Cas system of two *M. gallisepticum* isolates infecting poultry and house finch hosts. Using *in vitro* and *in vivo* approaches, we determined the PAM specificities of the MgCas9 and showed a correlation between a modification of the PAM signature, the evolution of the spacer repertoires and the evolution of the PAM-interacting (PI) domain of MgCas9. Such radical change in the CRISPR-Cas defence system of *M. gallisepticum* may have implications for the rapid adaptation of *M. gallisepticum* to its novel host [20,21].

## Methods

### Oligonucleotides and plasmids

All oligonucleotides used in this study were supplied by Eurogentec and are described in Table S1. All plasmids constructed and used in this study are listed in Table S2. Detailed protocols for plasmid construction are provided in SI-1.

### Genome sequences

The genomes of 79 *M*. *gallisepticum* isolates collected in house finch from 1994 to 2015 were sequenced using Illumina technology, and draft assemblies were produced by C. Bonneaud *et al*. as part of a global comparative genomic study (unpublished). All isolates were cultured in SP4 media supplemented with 0.1% phenol red (Sigma) as a growth indicator. Cultures were grown to mid-exponential phase as determined by the colour shift of the media from pink/red to orange. Genomic DNA was extracted and sent to the Earlham Institute (Norwich) where they were used for the construction of 250 bp paired-end libraries using the Low Input Transposase Enabled library preparation pipeline. The libraries were subsequently sequenced on an Illumina platform, with the VA 1994 isolate additionally sequenced using PacBio technology to produce a high-quality *de novo* genome assembly (Weinert et al., in prep). Genomes were annotated using Prokka (version 1.14.6) available at https://usegalaxy.eu. The CRISPR-Cas loci including *cas* genes, tracrRNA and arrays of spacers were extracted from the *M. gallisepticum* genomes for further comparative analyses. Their sequences are available in GenBank with accession numbers listed in Table S3. Other genomes used in this study were retrieved from GenBank, and their accession numbers are available in Table S3.

### Phylogenetic tree of MgCas9 and comparison of MgCas9 from S6 and CA06 isolates

Phylogenetic analysis of MgCas9 was performed using protein sequences and the phylogeny pipeline available at http://www.phylogeny.fr/. This pipeline includes the software muscle, Gblocks, PhyML and TreeDyn. The representation of the tree was designed on iTOL (https://itol.embl.de/). For the truncated forms of MgCas9, the different parts of the ORF were artificially merged to include them in the tree. Protein alignments were analysed on mega-x using muscle alignment software [22]. The mutation rates between S6 and CA06 were calculated from SNP detected using whole-genome nt alignment in MAUVE [23]. For mutation rates of proteins, concatenated protein sequences of whole genome were compared using blast-p.

### Whole proteome comparison of S6 and CA06 isolates

Proteome comparison between S6 and CA06 isolates was performed using the PATRIC tool (https://www.bv-brc.org/app/SeqComparison) and fasta protein files from NCBI. The settings used are as follows: minimum per cent coverage, 50%; blast *E* value 1e-5; and minimum per cent identity: 50%.

### *In silico* analyses of *M. gallisepticum* spacers

For each *M. gallisepticum* genome, spacers were detected using CRISPRFinder (https://crispr.i2bc.paris-saclay.fr/Server/) (database version: 2017-05-09). Draft assemblies obtained from Illumina short reads for newly sequenced isolates could contain uncertain nucleotides N. All spacers containing N tracks were removed to avoid an artificial increase of the spacer diversity. Using a homemade script, a matrix representing the occurrence of spacers in the *M. gallisepticum* genomes was produced (Table S4A). The 534 non-redundant spacers were next clustered by coverage and identity (≥ 90% each) using CD-HIT [24], and a new simplified matrix representing the occurrence of the 490 merged spacers was produced (Table S4C). PHASTER (update: 29 March 2018) [25], GenBank and IMG (Integrated Microbial Genomes) [26] databases were next requested to identify putative targets (blastn ≥90% for identity and coverage) (Table S4D–F). Spacers were also used in blastn queries against *M. gallisepticum* genomes; spacers with more than one hit were also listed (Table S4G).

### Construction of a minimal *M. gallisepticum* sgRNA

Sequences of DR and tracrRNA were concatenated, and the secondary structures of the hybrids were simulated using the mfold software at http://unafold.rna.albany.edu/. The sgRNA was designed according to Jinek *et al.* [27]. The chimeric molecule was synthesized by IDT DNA company and further cloned in the plasmid pET28.

### Bacterial isolates and culture conditions

*M. gallisepticum S6* (Tax ID: 1006581) was cultivated in modified Hayflick’s medium [28] under a 5% CO_2_ atmosphere. Tetracycline 5 µg ml^−1^ was used in selective conditions. *M. gallisepticum* S6 isolate with a modified PI domain of MgCas9 was obtained as described in [29].

### *In vitro* determination of MgCas9 PAM specificity

A 7N degenerated PAM plasmid library was provided by Dr Gasiunas from Caszyme [30]. Randomness of the seven positions of the randomized PAM was validated as described in [31]. *In vitro* cleavage assays were performed as explained in [30]. Details of the protocol are provided in SI-1.

### Transformation of *M. gallisepticum*

*M. gallisepticum* cells were grown for 36 h before transformation. At pH 6.2–6.5, aliquots of 10 ml were centrifuged for 15 min at 6000 ***g***, 10 °C. The cells were then resuspended with 5 ml of HBSS (Hanks’ Balanced Salt solution) 1× wash buffer (Thermo Fisher, 14065056) and centrifuged 15 min at 6000 ***g***, 10 °C. The pellet was resuspended in 250 µl of CaCl_2_ 0.1 M and incubated for 30 min on ice. Cold CaCl_2_-incubated cells were gently mixed with plasmid DNA (10 µg) and 10 µg of yeast tRNA (Thermo Fisher, AM7119). Then, 2 ml of 40% PEG 6000 (Sigma, 11130) dissolved in HBSS 1× buffer was added to the cells. After 2 min of incubation at room temperature, contact with the PEG was stopped by the addition of 20 ml of HBSS 1× wash buffer. The mixture was centrifuged 15 min at 6000 ***g***, 10 °C, and the cells were resuspended in 1 ml of modified Hayflick’s medium pre-warmed at 37 °C. After 2 h of incubation at 37 °C, the cells were plated on Hayflick selective plates. After 10–15 days at 37 °C with 5% CO_2_, colonies were counted, picked and resuspended in 1 ml of modified Hayflick’s medium with selection for three passages (one passage ~48 h).

## Results

### Comparison of the CRISPR-Cas locus across *M. gallisepticum* isolates

**Fig. 1.**
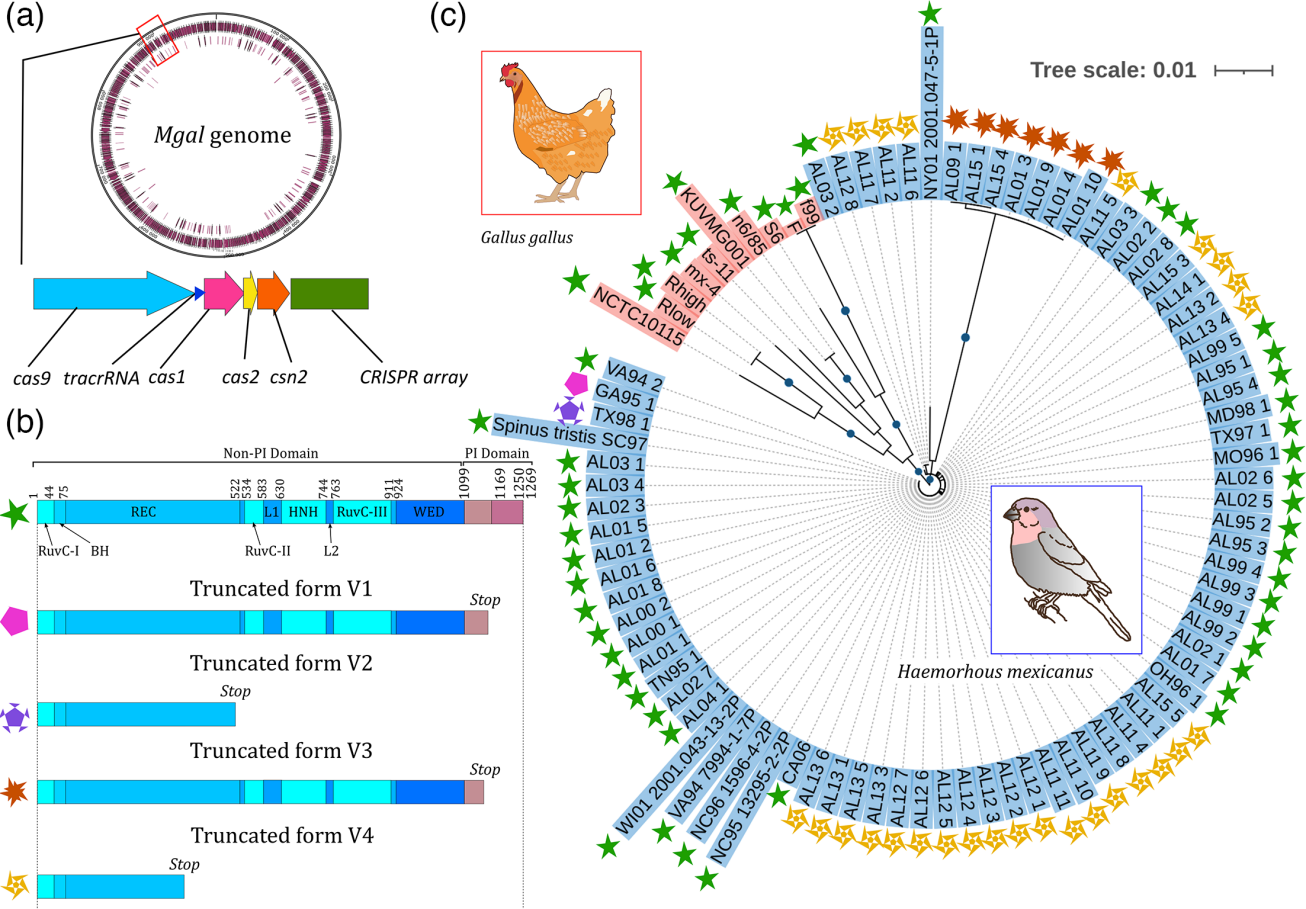
Presence and integrity of the CRISPR defence system in *M. gallisepticum* isolates. (a) Map of the *M. gallisepticum* genome highlighting the position of the CRISPR-Cas locus (red box) and its organization. (**b**) Domains present in full-length and truncated forms of MgCas9 found in *M. gallisepticum* isolates. Green star, isolates with a complete CRISPR locus and a complete MgCas9; other motifs, inactivated CRISPR system with truncated forms V1, V2 and V3 of MgCas9. (**c**) Phylogenetic tree of MgCas9. Isolates from poultry and house finch hosts are highlighted in red and purple, respectively. MgCas9 forms are indicated for all isolates. Green dots on branches indicate bootstrap values >95%.

CRISPR-Cas sequences were retrieved from the previously published whole-genome sequences of 19 poultry and 8 house finch strains [11,32,36], as well as from a further 79 newly sequenced genomes of isolates collected in house finches between 1994 and 2015 (Table S3). A single CRISPR locus was found in all *M. gallisepticum* isolates obtained from house finches (Fig. 1a), with the exception of six isolates (one collected in 2006, three in 2007, one in 2008 and one in 2015). In all six of these isolates, the CRISPR-Cas system was found to have been inactivated due to the loss of part of the locus harbouring the tracrRNA (Trans-activating crRNA) and the *cas* genes (i.e. *cas9*, *cas1*, *cas2* and *csn2*), leaving only the CRISPR array. An extended analysis of the genome region on both sides of the CRISPR locus further suggested this system being part of a larger defence island, also including a restriction–modification system, a toxin–antitoxin system [37] and a putative S8 serine peptidase (SI-2). For at least the two isolates with a completely assembled genome (isolates collected in 2006 and 2008), the deletion was extended upstream of the CRISPR locus, including the putative S8 serine peptidase and other adjacent genes. The inactivation of the CRISPR-Cas system could also be predicted for a further subset of isolates based on several mutations found in their MgCas9 gene. Indeed, we were able to predict four different truncated versions of MgCas9 (thereafter named V1, V2, V3 and V4) (Fig. 1b, Table S3). The V1 form was detected in one single house finch isolate sampled in 1995; with C to T substitution at position 3571, leading to a TAA stop codon and a MgCas9 protein truncated of 79 aas in the C-terminal. The MgCas9-truncated V2 form was also observed only once, in a 1998 house finch isolate; it is truncated at position 1498 in the REC domain as a result of a single base change that generates a TAG STOP codon instead of CAG Gln codon. The V3 form was detected in seven isolates sampled in house finches in 2001, 2002, 2009 and 2015; it was predicted based on a frameshift at position 3445 of the coding sequence (CDS), leading to a protein truncated of the last 120 aas within the PI domain. Finally, the V4 form was identified in 28 isolates collected in house finches between 2011 and 2015; it is truncated at position 1018 of the CDS within the REC lobe (which exerts a key role for DNA cleavage) as a result of a single nt mutation changing a GAA Glu codon into a TAA STOP codon. Additional mutations inactivating Cas1 or Cas2 were observed in isolates AL15_1, AL15_4 (inactivated MgCas9 form V3) and AL11_7 (inactivated MgCas9 form V4), respectively.

The pattern of occurrence of active and inactive MgCas9 in house finch isolates suggests an overlap of both forms over a relatively short period and followed by the complete inactivation of MgCas9. Indeed, all the house finch isolates for which we could predict a complete MgCas9 were collected between 1994 and 2006, whereas all the isolates collected after 2007 displayed either an inactivated MgCas9 or a partial deletion of the CRISPR locus. Isolates with active and inactive MgCas9 were therefore only found to co-occur in house finches between 1995 and 2006. This gradual pattern of inactivation contrasts with what we see in poultry, as all isolates collected in chicken and turkeys >60 years (~1958–2019) are predicted to harbour an active CRISPR system and did not display any inactivating mutations in MgCas9.

### Analysis of spacers and identification of *M. gallisepticum* CRISPR targets

We compared the set of spacers inferred from the *M. gallisepticum* CRISPR locus across all studied *M. gallisepticum* isolates (Fig. S1, available in the online version of this article, Table S4) and compiled a dataset of 490 non-redundant spacers (Table S4C). As a result, we identified a total of 438 unique spacers from 19 poultry isolate and 55 unique spacers from 85 house finch isolates (Table S4C). Across the *M. gallisepticum* poultry isolates, we observed a wide diversity of spacers, with no spacer found conserved across all isolates (Fig. S1). Indeed, 66% of spacers from the 19 poultry isolates were present in only 1–2 (0–10%) genomes, while 2% were found in 9–10 (40–50%) genomes. In contrast, we found a lower diversity of spacers across the CRISPR arrays of house finch isolates. A quarter (24%) of spacers found in house finches were shared across more than 80% of isolates, while over half (58%) were present in at least 40% of them, and 21 out of 55 (38%) spacers were present in 10% or less of house finch isolates.

We found only three spacers that were shared across poultry and house finch isolates (Table S4C). Spacer SP_516* was identified in the poultry vaccinal strain ts-11, in seven derivates obtained by chemical mutagenesis (K6372, K6356, K6212B, K6216D, K6208B, K5322C and K2966) and in all but ten of the house finch isolates. Spacer SP_119* was found in four ts-11 derivate strains (K6369, K6208B, K5322C and K6222B) and in three house finch isolates for which the CRISPR-Cas9 system was predicted to be inactive (169125442_2001, 13_09_02_NC and 2015_J). Finally, spacer SP_325* was shared between the poultry strains R_Low_ and R_High_ and by five house finch isolates (NC95, NY01, WI01, NC06 and NC08). When considering the pattern of spacer acquisition over time in house finches, we found that most new spacers (i.e. 46 out of 55; >80%) had been integrated before 2000 and were therefore present in the early years of *M. gallisepticum* emergence in house finches. Surprisingly, despite the inactivation of MgCas9 since 2007, we also found evidence for the acquisition of six new spacers between 2009 and 2015 (Table S4C).

We investigated the potential origin of *M. gallisepticum* spacers using blast queries against PHASTER, GenBank and IMG databases (Table S4D–G), as described in [38]. Out of 490 spacers, 431 (88%) showed no hits in the explored databases. The PHASTER database returned hits on known bacterial phages for 34 spacers. GenBank returned hits on bacterial genomes (11 spacers) and phages (4 spacers). IMG returned hits on bacterial genomes for eight spacers. In addition, two self-matching spacers were found in two strains of *M. gallisepticum* (ts-11 and NTCTC1015). Overall and across the 57 spacers with identified hits outside *M. gallisepticum* genomes, we noted 6 spacers that were present in house finch isolates and 51 in those from poultry. Out of the 19 spacers with hits on bacterial genomes, 10 returned non-CRISPR genomic regions of mycoplasma. Among them, six spacers from poultry strains (S6, KUVMG001, 6/85, ts-11 and ts-11 derivates) matched to the genome of *Mycoplasma imitans* (also named *Mycoplasmoides imitans*), a duck pathogen phylogenetically related to *M. gallisepticum* (Table S4F); these potential protospacers are localized in a *M. imitans* chromosomal region of 14 kb that is not found in the *M. gallisepticum* genomes used in this study, contrary to other surrounding loci. In this region, we identified several genes considered essential for the mobilization and transfer of mycoplasma integrative and conjugative elements (MICEs) (Fig. S2) [39,41]. This suggested that those six spacers might be directed against a potential mobile element of 14 kb from bird mycoplasma, although no trace of this element has been detected in the *M. gallisepticum* genomes studied here. Taken together, these results indicate that *M. gallisepticum* CRISPR-Cas9 systems are mostly directed against unknown targets and that predicted targets include most predominantly phages, as well as a newly identified ICE-like (Integrative and Conjugative Elements) element of a bird mycoplasma.

### Diversity and evolution of Cas proteins

**Fig. 2.**
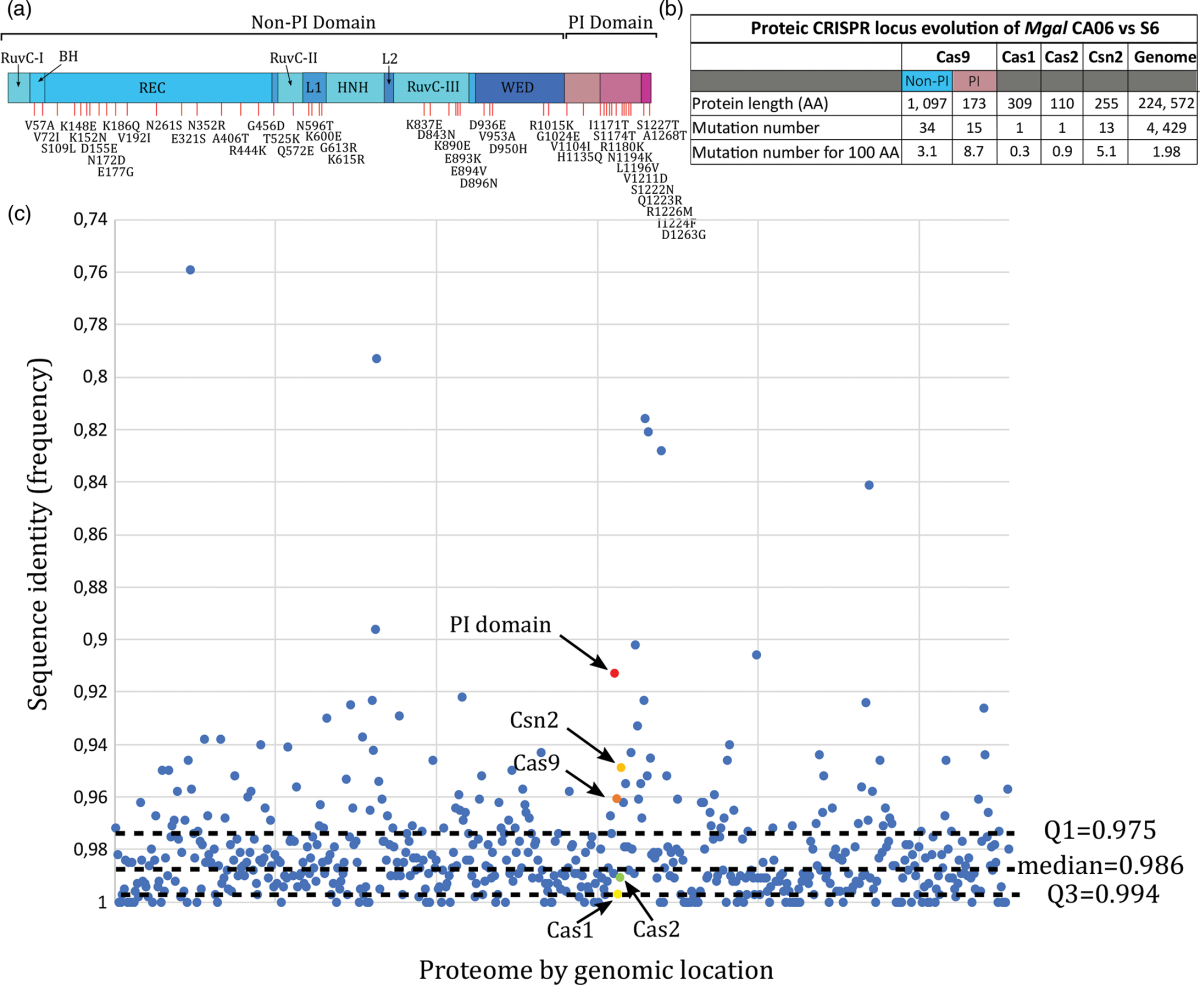
Evolution of the Cas proteins from *M. gallisepticum* poultry and house finch isolates. (a) Representation of MgCas9 protein variable positions (red dash) between S6 (poultry) and CA06 (house finch) isolates. For each mutation, the corresponding aa substitution is indicated. (**b**) Protein sequence comparisons between CRISPR effectors of S6 and CA06 isolates. (**c**) Protein identity plot. Percentages of identity are represented in the graph for each protein found in *M. gallisepticum* S6 genome and CA06 genome from PATRIC analysis. Each protein pair is represented as a coloured dot. *X*-axis corresponds to the genome location of the corresponding gene on the S6 genome; *Y*-axis indicates the sequence identity between the homologous proteins.

We undertook further *in silico* analyses to document the intraspecific diversity of *M. gallisepticum* Cas proteins across isolates displaying a complete functional locus. To do so, we aligned the protein sequences of MgCas9 and constructed a phylogenetic tree (Fig. 1c). The isolates were clearly distributed into two main branches, with a clustering of the poultry MgCas9 sequences in one branch and of the house finch ones in another. House finch MgCas9 sequences were found to be >99% identical to each other, and the copy present in the CA06 isolate was selected (as in [30]) to be compared with a poultry MgCas9 from the S6 isolate. This isolate was chosen because it has not been mutagenized and it has been proven to be transformable, which was required for further experimental approaches. While being 96% identical in sequence, the highest proportion of aa variations was found within the PI domain, which is involved in the interaction with the PAM sequence (Fig. 2a). Indeed, sequence variations of 8.7 and 3.1% were calculated for the PI domain and the other domains of the protein, respectively.

When considering other Cas proteins encoded within the CRISPR locus, we found that Csn2 sequences exhibited more variation (5.1%) than either Cas1 (0.3%) or Cas2 (0.9%) (Fig. 2b). A proteome-wide comparison of *M. gallisepticum* CA06 and S6 isolates showed that Cas9 and Csn2 are part of the 12% most divergent proteins between the two isolates (Fig. 2c, Table S4). Together, our results suggest that the jump of *M. gallisepticum* from poultry into house finches has been marked by a noticeable evolution of the MgCas9 PI domain (thereafter called MgCas9 PI domain) and of Csn2, both of which are key proteins in the ability of CRISPR-mediated immunity to acquire new spacers [18,19]. Since the PI domain of Cas9 is involved in PAM recognition, our results further suggest that the jump might have been associated with a change in the PAM recognition specificity of MgCas9, a pattern which is consistent with a change in pathogen pressure experienced by *M. gallisepticum* within the novel finch host.

### *In vitro* PAM specificity differs between poultry (S6) and house finch (CA06) MgCas9

**Fig. 3.**
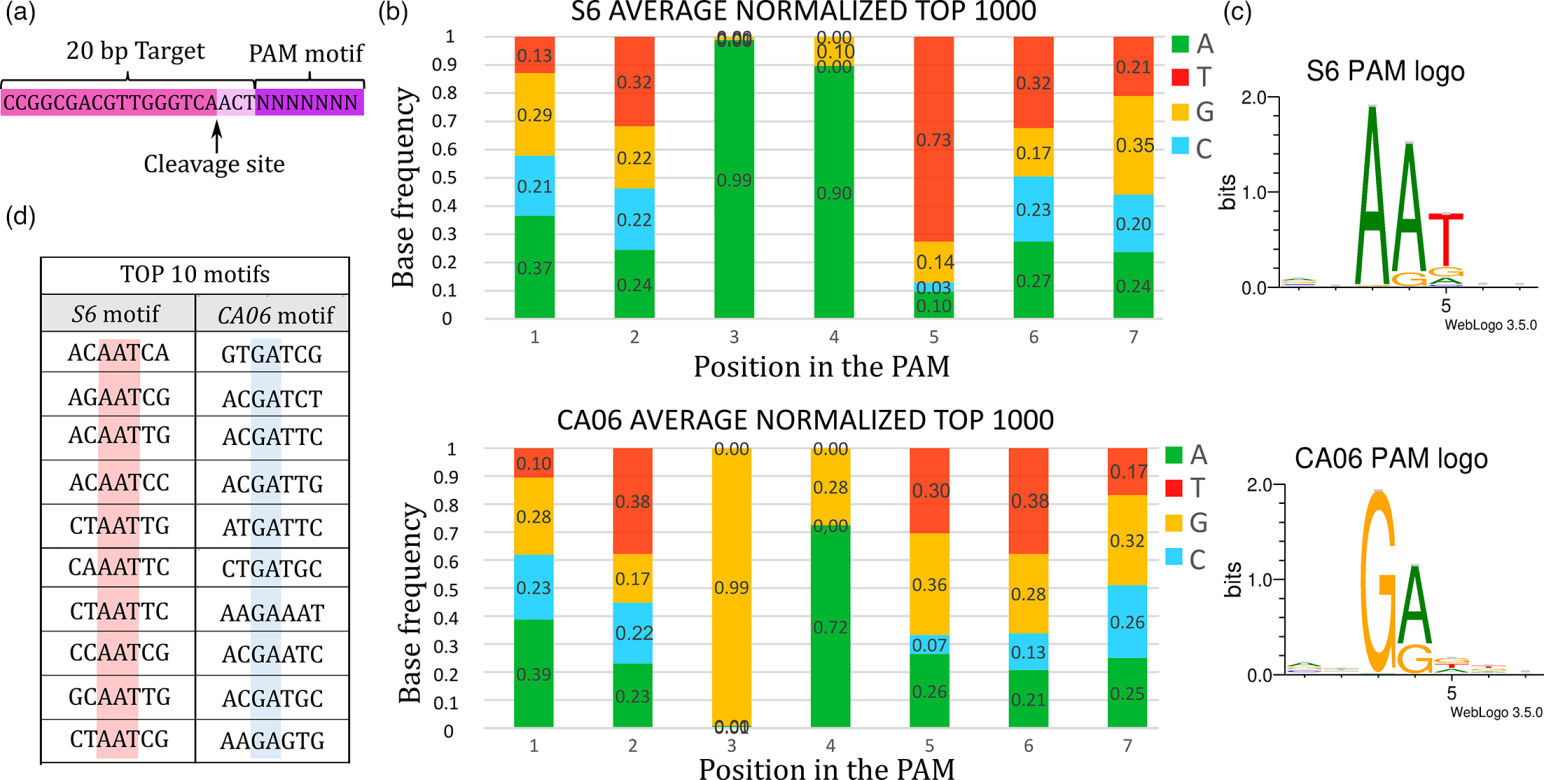
*In vitro* characterization of PAM sequences recognized by MgCas9 from poultry (S6) and house finch (CA06) isolates. (a) Position of the observed cleavage site for the two MgCas9. (**b**) Position frequency matrix (PFM) observed after cleavage of a 7-position PAM-degenerated library by MgCas9 from S6 and CA06 *M. gallisepticum* isolates. Frequencies were averaged on three replicates and corrected considering the bias in the distribution of PAM sequences in the initial plasmid library. Adenine is represented in green, cytosine in blue, guanine in yellow and thymine in red. Frequencies of each base at each of the seven positions are indicated on the graph. (**c**) Web logos of consensus PAM sequences recognized by MgCas9 S6 or CA06. (d) Top ten of the best motifs recognized *in vitro* by MgCas9 S6 and CA06.

We used an *in vitro* assay previously developed [30,31] to compare the PAM recognition preference of MgCas9 between the poultry isolate S6 and the house finch isolate CA06. *In vitro*-produced MgCas9/sgRNA (single guide RNA) ribonucleoparticles were used for the cleavage of a library of plasmids containing a target sequence and a PAM sequence randomized on seven positions (Figs 3a and S3, Table S6). After three independent *in vitro* cleavage of the plasmid library by MgCas9 S6 or MgCas9 CA06, molecules containing a recognized PAM sequence were captured using DNA ligation of adapters, enriched by PCR amplification and submitted for deep sequencing. Cleavage was observed three bases upstream from the PAM sequence at >99.95%, and the results obtained for replicates of the two series were nearly identical (Fig. S4A, B). Out of the 16 306 PAM sequences contained in the library, 10 928 and 9709 PAM sequences were detected at least once after cleavage by MgCas9 S6 and MgCas9 CA06, respectively. The 1000 most frequent PAM sequences were selected for each MgCas9 including 74 PAM sequences that were common to both MgCas9 S6 and MgCas9 CA06 top 1000 PAM sequences. Normalized frequencies were calculated, taking the bias of the original plasmid library into account (Table S6F); frequency matrices and sequence logos were produced (Fig. 3b, c, Table S6G).

A comparison of the PAM recognition preference of MgCas9 S6 and MgCas9 CA06 revealed that the PAM sequences recognized by both correspond to a five-letter motif, with a marked bias observed at positions 3, 4 and 5 only. Indeed, two lines of evidence indicate that the 5-position PAM sequences recognized by MgCas9 from poultry S6 and house finch CA06 isolates differ *in vitro*. First, a sequence logo comparison revealed that the PAM preferences of MgCas9 proteins were clearly different between the two isolates. The main difference was observed at position 3 with 99% of sequences displaying an A in MgCas9 S6 and a G in MgCas9 CA06 (Fig. 3b). Position 4 was preferentially A or G in both cases, with a bias towards A more marked in MgCas9 S6. In position 5, a T was present in 73% of sequences retrieved after cleavage by MgCas9 S6, compared to 30% by MgCas9 CA06, suggesting a notable difference at this position between the two isolates. Second, we found that the top ten PAM sequences most frequently recognized differed between S6 and CA06 MgCas9 (Fig. 3d).

To confirm the difference in PAM recognition specificity, we selected two PAM sequences, specifically recognized by MgCas9 S6 (motif 1, AT**AAA**AA) and MgCas9 CA06 (motif 2, AA**GAG**AA), respectively. Three plasmids were constructed containing the target sequence followed by one of the two selected PAM sequences or by the seven first nts of direct repeat (DR) sequence interspacing spacers in *M. gallisepticum* CRISPR arrays (motif 3, GT**TTT**AG) (Fig. S5A). After *in vitro* cleavage of these plasmids by *in vitro*-produced MgCas9/sgRNA complexes, we observed that MgCas9 S6 efficiently cleaved motif 1 but not motif 2, whereas MgCas9 CA06 preferentially cleaved motif 2 (Fig. S5B). By contrast, control motif 3 remained uncleaved after incubation with one MgCas9 or the other. This confirmed the difference in PAM specificity of MgCas9 originating from poultry and house finch isolates, *in vitro*. Together, our results show that poultry and house finch MgCas9 recognize different five-letter motifs in the PAM sequences and display different cleavage specificities of PAM sequences as a result.

### *In vivo* PAM specificity of MgCas9 is driven by PI domain evolution

**Fig. 4.**
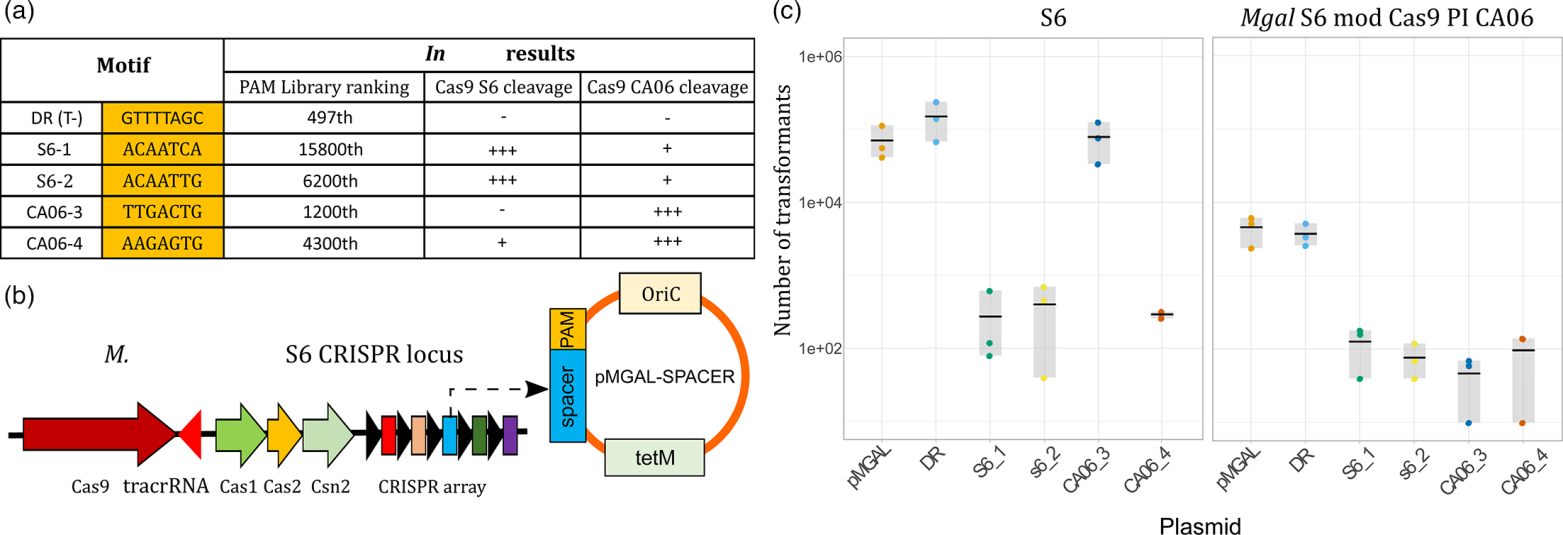
Evaluation of MgCas9 PAM specificity *in vivo* using a plasmid interference assay. (a) PAM sequences for *in vivo* interference assays were selected from *in vitro* results. DR is the DR sequence naturally present in the *M. gallisepticum* CRISPR array; this motif cannot be recognized by MgCas9 and is used as a positive control of transformation and negative control for cleavage. Four other PAM sequences were chosen, which are differentially cut *in vitro* by MgCas9 S6 and CA06. PAM sequences frequently retrieved after cleavage are noted ‘+++’, whereas lowly and not recognized sequences are indicated by ‘+’ and ‘-’, respectively. (**b**) Plasmid design for interference assays in *M. gallisepticum* S6 and *M. gallisepticum* S6 mod PI CA06. Plasmid pMgal contains *M. gallisepticum oriC*, a *tet(M)* resistance marker and the third spacer, which is present in the S6 CRISPR array, followed by selected PAM sequences. (**c**) *In vivo* result of transformation assays for the two *M. gallisepticum* strains. The numbers of transformants obtained after three transformation replicates are represented as a dot plot.

We characterized the PAM specificity of poultry (S6) and house finch (CA06) MgCas9 using *in vitro* and *in vivo* approaches. Based on the results previously obtained *in vitro*, we selected four PAM motifs found to be differentially cut by poultry (S6) and house finch (CA06) MgCas9 (Fig. 4a). Motifs S6-1 and S6-2 were found to be recognized by MgCas9 S6 *in vitro*, but poorly by MgCas9 CA06*,* while motif CA06-3 was cleaved by MgCas9 CA06 but not MgCas9 S6, and CA06-4 was efficiently recognized by MgCas9 CA06 but only weakly by MgCas9 S6. To investigate whether these findings obtained *in vitro* are representative of processes occurring *in vivo*, we conducted interference assays on both the WT *M. gallisepticum* S6 isolate and on a genetically engineered *M. gallisepticum* S6 strain (thereafter named *M. gallisepticum* S6 mod PI CA06) in which the MgCas9 PI domain was seamlessly replaced by the homologous domain of the MgCas9 CA06 [29]. The *in vivo* cleavage assay was based on the transformation of *M. gallisepticum* with a replicative plasmid containing the third spacer naturally found in the CRISPR array of isolate S6, followed by a selected PAM candidate (Fig. 4b). A plasmid that contained the DR of the CRISPR array as a PAM sequence was used as a negative control.

Transformations with plasmids containing PAM sequences expected to be cut by MgCas9 S6 (S6-1 and S6-2) led to 100–1000-fold less transformants compared to the transformation with the control plasmid (Fig. 4c). This confirmed that the CRISPR-Cas9 system is functional as a defence system in *M. gallisepticum* S6 and that PAM sequences recognized *in vitro* by MgCas9 S6 are also recognized *in vivo*. Similarly, plasmids harbouring PAMs frequently cleaved by MgCas9 CA06 *in vitro* were also cleaved by MgCas9 mod PI CA06 *in vivo*, demonstrating that the hybrid MgCas9 is fully active. Interestingly, in this interference assay, motifs that were poorly recognized *in vitro* (i.e. CA06-4 for MgCas9 S6 and S6-1 and S6-2 for MgCas9 CA06) were efficiently cleaved *in vivo* (see SI-3 for further comparisons of *in vitro* and *in vivo* findings). Finally, as we expected, we found that the PAM CA06-3 was not recognized by MgCas9 S6 but was efficiently cleaved by the hybrid MgCas9 that included a CA06 PI domain. This result confirms that the specificity of MgCas9 is mediated by the PI domain and suggests that mutations in the MgCas9 of house finch isolates have led to a change in PAM specificity.

## Discussion

We studied the genomes of *M. gallisepticum* isolates that were collected in the original poultry host and from house finches periodically over a 21-year span following a host shift. Our focus was investigating the impact of a dramatic change in the host environment on the evolution of the pathogen’s CRISPR-Cas defence system. First, our results were consistent with a previous observation, made from a small number of isolates, that there has been a gradual inactivation of MgCas9 in the house finch host since colonization. Indeed, we found that, while all poultry isolates displayed an active CRISPR-Cas system, both active and inactive forms of MgCas9 were present in house finch isolates collected in the initial 12 years following the host shift. After the twelfth year following colonization, however, all *M. gallisepticum* collected from house finches displayed either an inactivated MgCas9 or a partial loss of their CRISPR locus (*cas* genes). Second, of the 490 unique spacer sequences detected across poultry and house finch isolates, only 3 were shared across both avian hosts, with poultry isolates displaying a greater diversity of sequences than house finch isolates. Third, we found that the protein sequences of MgCas9 of poultry and house finch isolates clustered into two distinct phylogenetic branches, with very little variation detected among house finch MgCas9 protein sequences. Within MgCas9, the PI domain (involved in the specific interaction between Cas9 and the PAM that follows the target DNA sequence) and the Csn2 protein sequences were found to be most variable across poultry and house finch isolates. Fourth, we found that MgCas9 recognizes a five-letter motif in the PAM sequence. The motif recognized differed between poultry and house finch MgCas9, and, accordingly, we measured the variation in cleavage efficiencies *in vitro*. The poultry MgCas9 was more effective at cleaving its target PAM sequences than the house finch MgCas9, while the house finch MgCas9 was most effective at cleaving its target PAM. Finally, our results confirmed that the differences in the PAM specificity of MgCas9 are driven by the differences in the PI domain between poultry and house finch isolates. Indeed, the differences in specificities were replicated across a WT poultry isolate (S6) and a genetically engineered mutant of that isolate in which the PI domain of MgCas9 was replaced by the homologous domain of a house finch isolate (CA06), with the latter more effective at cleaving PAMs from house finch isolates. Together, these results suggest that significant evolutionary changes took place in *M. gallisepticum*’s CRISPR-Cas defence system following the jump into the novel house finch host. We documented an initial pattern of acquisition and loss of CRISPR spacers and of changes in PAM specificity driven by the MgCas9 PI domain. This observation is consistent with an evolutionary response to exposure to a new community of phages and MGEs. That such initial functional changes were followed by the subsequent inactivation of this defence system, however, highlights the complexity of the selection pressures that it faced.

Our study on the CRISPR-Cas9 system of *M. gallisepticum* isolates collected in both poultry and house finch hosts provides support for previous work, while also broadening our understanding of the complexity of this system across vertebrate host environments. First, mycoplasma CRISPR-Cas9 systems have previously been classified as type II *in silico*, which include the typical set of *cas* genes, *cas1*, *cas2*, *cas9* and *csn2* [16]. Accordingly, we found that the CRISPR-Cas systems of *M. gallisepticum* poultry S6 and house finch CA06 isolates are typical of the type II systems. Second, our results are also consistent with a previous study that undertook the first functional investigation of the Cas9 endonuclease in a mycoplasma system as part of a larger initiative to characterize 79 Cas9 orthologues representative of ten major clades in a Cas9 evolutionary tree [30]. In this study, the house finch isolate CA06 was selected as representative mycoplasma Cas9 with a 5-position motif NNGAD as its consensus PAM [30]. Our results provide confirmation for MgCas9 CA06’s consensus PAM. Interestingly, however, we identified a different PAM motif (NNAAT) in the MgCas9 of the poultry isolate S6, which was found to exhibit an ultra-dominant A at position 3 (99%) and a marked trend (73%) towards a T at position 5. In addition, we found that this difference in the PAM recognition preference of MgCas9 was associated with differences in both the composition and diversity of the spacers between poultry and house finch isolates, suggestive of a complete reset of the CRISPR array following the host shift.

An active adaptive immunity system like CRISPR-Cas is expected to be maintained only when the benefits of such a system outweigh the costs of sustaining it [4,6, 42]. The main benefit of an active CRISPR-Cas system is the protection that it confers against phages and MGEs. For example, in the case of *M. gallisepticum*, the presence in poultry isolates of six spacers matching to the genome of the duck pathogen *M. imitans* suggests that avian mycoplasma bacteria are under selection to actively protect themselves against invasion by circulating MICEs. However, maintaining an active CRISPR-Cas undoubtedly incurs fitness costs. Such costs can arise, for instance, when CRISPR-Cas generates metabolic costs [43,44] and genetic conflicts [43,44] or when it is less effective at preventing infections than other defence processes [45,47]. CRISPR-Cas can also create a risk of self-targeting spacers [46,48] and can prevent the acquisition of fitness-enhancing genes through HGT [6,49,56]. The balance between the costs and benefits of maintaining an active CRISPR-Cas will therefore depend on CRISPR-Cas’ contribution to the bacterium’s global defence process, on its non-defence roles and on the specific selective pressures exerted by different host environments.

**Fig. 5.**
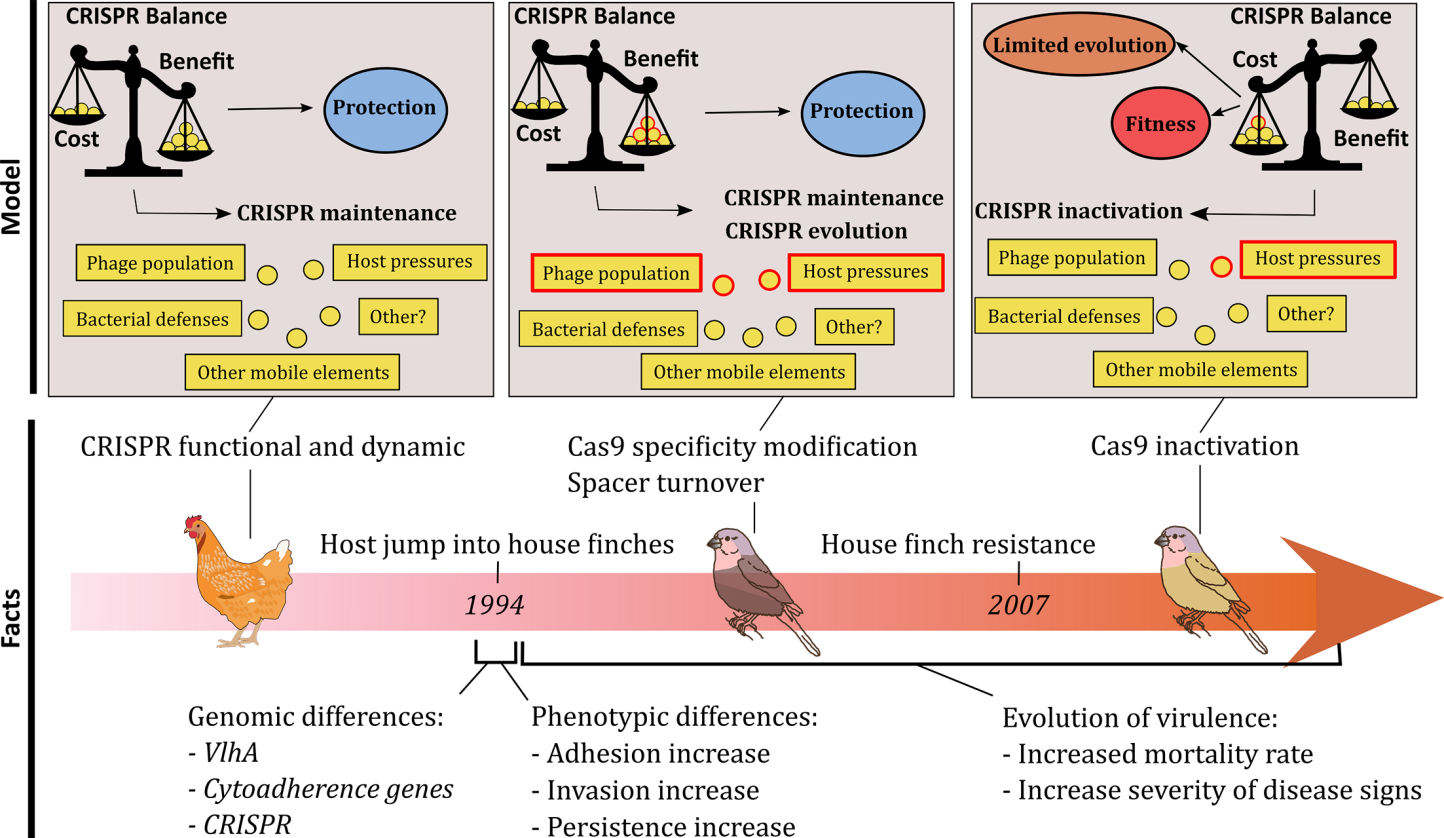
Hypothetical model of the evolution of the CRISPR-Cas system in *M. gallisepticum* as it jumped from poultry and emerged into the novel house finch host. Phenotypic and genetic changes accompanying each step are obtained from the literature.

That there were isolates in circulation with an active (albeit reconfigured) CRISPR-Cas system in the 12 years following the jump into house finches indicates that there were benefits to the maintenance of this defence mechanism during that time period (Fig. 5). The retention of an active CRISPR-Cas system is particularly striking when one considers the function of the system was maintained even with a large number of mutations (49 aa changes when comparing MgCas9 S6 and MgCAs9 CA06) that accumulated in the house finch *cas9* gene following the jump. The reasons for such benefits are likely to be found in the distinct PAM specificity of MgCas9 in house finch isolates, alongside salient patterns of loss of ‘poultry-type’ spacers and acquisition of new spacer elements. Indeed, spacers that are no longer in use are expected to be lost, while new ones that confer resistance against novel pathogenic targets should be gained. *M. gallisepticum* isolates from house finches display dramatic changes both in the spacer set and MgCas9 PAM specificity, which suggest that the bacterium was faced with a massively different community of phages and invasive MGEs in the novel host. Indeed, such a finding is consistent with the previous evidence of a rapid evolution of PAM specificities reported to occur in response to an escape from detection by phages and MGEs displaying mutations in their PAM motifs [57,59]. Whether *M. gallisepticum* experienced novel pathogen-driven selection in house finches and the role that this might have played in facilitating or hindering its emergence remain to be determined. Regardless, our results give rise to the hypothesis that the successful emergence of a bacterial pathogen in a new host species will not only depend on its ability to establish and spread in the new host but also on its ability to cope with a new landscape of phages and other invading DNAs. Currently, no prophages have been described in *M. gallisepticum* genomes, while several phages and prophages have been characterized since the 1970s in some other Mollicutes, including *Acholeplasma*, *Spiroplasma* and *Mycoplasma* species [60]. In addition, in a recent survey of bacterial genomes and metagenomes, 101 prophages were predicted in *Mycoplasma*, 76 in *Spiroplasma*, 3 in *Mesoplasma*, 6 in *Ureaplasma*, 18 in *Ca*. phytoplasma and 132 in *Acholeplasma* and related bacteria [61]. The impact of phages on the evolution of Mollicutes remains largely unknown, but these data indicate that these bacteria, even those with an intracellular lifestyle (i.e. *Ca*. phytoplasma), regularly encounter phage attacks, which is in accordance with the presence of defence systems in most species. In this perspective, the growing use of metagenomics to explore bacterial and viral communities in poultry already evidenced some phages [62] and opens the way to future studies focusing on the phage repertoires of poultry and house finch infected or not with *M. gallisepticum*.

As *M. gallisepticum* adapted to its new host and novel sets of phages and invasive MGEs, the costs of maintaining an active CRISPR-Cas system seem to have outweighed the benefits of such a system. Isolates displaying an inactive CRISPR-Cas system were also found in circulation over the 12 years following the jump into house finches, and, by 2007, all the isolates collected displayed either an inactivated MgCas9 or the complete loss of their *cas* genes locus, leaving only the CRISPR array (Fig. 5). Indeed, *cas9* inactivation evolved at least four times over that period from different point mutations leading to stop codons. Additional mutations inactivating *cas2* or *cas1* were also observed in some 2011 and 2015 isolates, confirming a trajectory of degradation for the CRISPR-Cas system.

When considering the repertoire of defence systems predicted in the genome of *M. gallisepticum* (SI-2, Table S7), the inactivation of the CRISPR system in house finch isolates after 2007 was, however, not correlated with the acquisition of another, potentially compensatory system. This means that the loss of the CRISPR system is unlikely to have resulted from the acquisition of a less costly defence mechanism.

Across bacteria, the switching off of the CRISPR-Cas systems has nevertheless also been proposed as a mechanism by which to respond to selection [63]. In *Escherichia coli*, for example, CRISPR-Cas is thought to restrict adaptive change by limiting the acquisition of potentially beneficial genes through horizontal transfer, giving rise to a trade-off between defence and adaptive potential [64]. As a result, we might expect the balance of costs and benefits of maintaining an active CRISPR-Cas system to have shifted for *M. gallisepticum* in the face of a significant change in selection in the novel host. This loss of function has not been associated with the acquisition of another defence system, as we only found the IetA/S system, which is stably maintained in all HF (House finch) and poultry strains. The putative S8 protease gene is also found in HF strains, except for those that have lost the entire CRISPR locus (Fig. S6, Table S7). Altogether, the overall trend for the bacteria is a general loss of the defence arsenal, suggesting less external pressure.

One major selective event faced by *M. gallisepticum* after the jump into house finches was the evolution of host resistance to infection [65,67]. The jump gave rise to an epidemic that spread quickly and is thought to have killed millions of house finches [17,68]. The high mortality rate of *M. gallisepticum* in house finches was a result of *M. gallisepticum* localizing in the mucosal surfaces of the conjunctiva and upper respiratory tract, causing severe conjunctivitis that can lead to death in the wild through blindness-induced starvation or predation [69,70]. The intensity of this selective event led to the spread of resistance in house finch populations from standing genetic variation and within only 12 years of epidemic outbreak [20,65]. As a result, by 2007, the proportion of resistant individuals in exposed house finch populations had risen to ~80% [65]. Such host resistance was, in turn, found to have significant selective consequences for *M. gallisepticum*. Indeed, resistance favoured the evolution of increasing pathogen virulence through antagonistic coevolution [20], with isolates causing ever greater host mortality and more severe signs of conjunctivitis over time [21], as well as transmitting faster to uninfected sentinels [71]. The concordance in the timing of the complete inactivation of CRISPR-Cas (i.e. from 2007) and the need for evolutionary changes in virulence in response to widespread house finch resistance is striking. Further work is now required to test whether significant phenotypic and genetic changes have, indeed, taken place following the loss of a functioning CRISPR-Cas, as predicted if this defence system hindered the necessary adaptive responses.

In conclusion, our study of the CRISPR-Cas defence system of bacterial isolates collected from the original host, as well as from a novel host into which it jumped recently, reveals marked differences between both in terms of the following: (1) the MgCas9 PAM recognition specificity, (2) the composition and diversity of the spacer repertoire and (3) the gradual inactivation of CRISPR-Cas over time in the novel host. While the changes in MgCas9 PAM specificity and in the composition and diversity of spacers indicate a change in the landscape of phages and MGEs faced by the bacterium in the novel host, the subsequent inactivation of CRISPR-Cas is consistent with another important selection event. We hypothesize that the gradual inactivation of the CRISPR-Cas defence system, which took place over the first 12 years of the epidemic until this defence system became fully inactivated from 2007, occurred in response to the evolution of host resistance, which became widespread by 2007 and pushed for adaptive changes in the bacterium. Such adaptive changes would have necessitated the loss of a functional CRISPR-Cas defence system to take place. Together, our results highlight the need to consider not only the host-driven selection pressures a bacterium experiences but also the complex interplay between phages and defence systems for a better understanding of the key factors driving the emergence of a pathogenic bacterium in a novel host.

## supplementary material

10.1099/mgen.0.001320 Uncited Fig. S1.

10.1099/mgen.0.001320 Uncited Table S1.

10.1099/mgen.0.001320 Uncited Table S2.

10.1099/mgen.0.001320 Uncited Table S3.

10.1099/mgen.0.001320 Uncited Table S4.

10.1099/mgen.0.001320 Uncited Table S5.

10.1099/mgen.0.001320 Uncited Table S6.

10.1099/mgen.0.001320 Uncited Table S7.

## Abbreviations

CDS: coding sequence
CRISPR: clustered regularly interspaced short palindromic repeat
DR: direct repeat
HGT: horizontal gene transfer
MgCas9: *M. gallisepticum* Cas9
MGEs: mobile genetic elements
MICEs: mycoplasma integrative and conjugative elements
PAM: protospacer adjacent motif
PI: PAM interacting
